# Macronutrient variability in human milk from donors to a milk bank: Implications for feeding preterm infants

**DOI:** 10.1371/journal.pone.0210610

**Published:** 2019-01-25

**Authors:** Ashley John, Ruichen Sun, Lisa Maillart, Andrew Schaefer, Erin Hamilton Spence, Maryanne T. Perrin

**Affiliations:** 1 Department of Industrial Engineering, University of Pittsburgh, Department of Industrial Engineering, Pittsburgh, Pennsylvania, United States of America; 2 Department of Computational and Applied Mathematics, Rice University, Houston, Texas, United States of America; 3 Mothers’ Milk Bank of North Texas, Fort Worth, Texas, United State of America; 4 Department of Nutrition, University of North Carolina Greensboro, Greensboro, North Carolina, United States of America; University of Illinois, UNITED STATES

## Abstract

**Background and objective:**

The composition of human milk varies widely and impacts the ability to meet nutrient requirements for preterm infants. The purpose of this study is to use a large dataset of milk composition from donors to a milk bank to: (1) describe the macronutrient variability in human milk and how it contributes to the ability to meet the protein and calorie targets for the preterm infant using fortification with commercially available multi-nutrient fortifiers; (2) assess how temporal versus subject effects explain macronutrient variability; (3) determine how macronutrient variability contributes to the nutrient distribution in pooled donor milk.

**Methods:**

This is a retrospective, observational study that analyzes the macronutrient data of 1,119 human milk samples from 443 individual donors to a milk bank. We test fortification strategies with potential basic, intermediate, and high protein and calorie commercial fortifiers. Additionally, we simulate the random pooling of multiple donors to model the impact of macronutrient variability on pooled donor milk.

**Results:**

Fat was the most variable nutrient and accounted for 80% of the difference in calories. A subject-effect predicted more of the variability after 4 weeks postpartum in all macronutrients (R^2^ > = 0.50) than a time-effect (R^2^ < = 0.28). When pooling multiple donors, variability was reduced by increasing the number of donors randomly selected for a pool or targeted pooling based on macronutrient analysis of donor pools. Over 75% of mature milk samples fortified with a basic protein fortifier did not meet daily protein targets of 3.5 g/kg without exceeding volumes of 160 ml/kg/day.

**Conclusion:**

There is a strong individual signature to human milk that impacts the pooling of donor milk, and the ability to meet protein and energy requirements for the preterm infant with basic and intermediate protein and calorie fortifiers.

## Introduction

Growth velocity is frequently monitored in the neonatal setting due to evidence that adequate growth in preterm infants is associated with improved neurocognitive development [1]. Higher nutrient intake has been shown to improve neurodevelopment outcomes [2,3] and reduce adverse events including necrotizing enterocolitis (NEC), late onset sepsis and bronchopulmonary dysplasia [4]. Increasingly, human milk feeding is recognized as an important strategy for reducing the incidence of NEC among preterm infants, though nutrient fortification is typically required to support adequate in-hospital growth [5–9], with protein often implicated as the rate-limiting macronutrient in human milk [10,11].

The underlying variability in human milk must be considered when trying to meet the nutrient needs of the preterm infant. Several trends in the macronutrient composition of human milk are well described in the literature: a decline in protein and an increase in fat content during the early days following parturition [12,13]; greater protein content in preterm versus term milk up to 2 months postpartum [14,15]; within feed differences in fat composition, with hind milk significantly higher in fat than fore milk [16,17]. Recent evidence suggests that an overall subject-effect explains more of the nutrient variability in mature human milk than a temporal effect [18], which may have significant implications for feeding the preterm infant.

Despite improvements in growth velocities in Very Low Birth Weight (VLBW) infants (less than 1500 grams), a study of over 360,000 VLBW infants between 2000 and 2013 found that 50% were classified as growth restricted at hospital discharge, defined as falling below the 10th percentile of weight for postmenstrual age on the Fenton growth chart [19]. The purpose of this study is to use a large dataset of milk composition from donors to a milk bank to: (1) describe the macronutrient variability in human milk and how it contributes to the ability to meet the protein and calorie targets for the preterm infant using fortification with commercially available multi-nutrient fortifiers; (2) assess how temporal versus subject effects explain macronutrient variability; (3) determine how macronutrient variability contributes to the nutrient distribution in pooled donor milk.

## Methods

This is a retrospective, observational study of the macronutrient composition of human milk provided by donors to the Mothers’ Milk Bank of North Texas (MMBNT; Fort Worth, TX) and received between November 2015 and July 2016. MMBNT follows the guidelines of the Human Milk Banking Association of North America (HMBANA) [20] and accepts milk after donors pass a verbal and serological screening. As part of the screening process, donors provide written, informed consent that their milk, or data about their milk, may be used for research.

### Milk samples and dataset

Approved donors collect and freeze expressed breast milk from individual pumping session in containers (e.g., plastic bags designed for human milk storage), typically in 1–4 ounce volumes, until they are ready to send the aggregate collection of milk to MMBNT for processing. When MMBNT receives a donor shipment, the frozen containers from an individual donor are sorted by pump date, grouped into one or more Deposits, and stored, frozen, until processing. MMBNT records the estimated volume, expiration date, and donor ID for each Deposit. The expiration date is set to the earliest pump date in the Deposit plus one year. During daily milk processing at MMBNT, for each donor selected, one or more frozen Deposits are thawed and aggregated into a Donor Pool. That is, Donor Pools can consist of the milk from single or multiple Deposits, representing a range of pump dates, all from the same donor.

Once each donor’s thawed milk is mixed into a Donor Pool, a sample of it is analyzed for fat, protein, and lactose using a MilkOScan FT 120 (Foss, Eden Prairie, MN). The MilkOScan uses Fourier transformed infrared spectroscopy (FT-IR) to measure macronutrients based on unique vibration patterns of chemical functional groups in each class of macromolecule. Precision and accuracy for measuring protein and fat in human milk using IR technology and the MilkOScan have been reported by others, though variability with lactose measurement was observed, likely due to interference from human milk oligosaccharides [21,22]. MMBNT calibrates the MilkOScan monthly using human milk standards that have been validated at a USDA facility (USDA & Marketing Services Dairy Program, Carrollton, TX) using enzymatic methods to quantify carbohydrates, the Kjeldahl method to quantify protein, and ether extraction to measure fat.

Macronutrient data for each Donor Pool is logged in MMBNT’s Timeless (Timeless Medical Systems, Henderson, KY, USA) database and a spreadsheet. Other information available in MMBNT’s dataset includes: Donor ID, Baby Date of Birth (DOB), Baby Term (full term, preterm, or deceased), Deposit Receipt Date, Deposit Expiration Date, and Deposit Volume.

### Computed values

Several values were calculated. For Donor Pools that contained a single Deposit, Pump Date was computed using Deposit Expiration Date less one year. For Donor Pools with multiple Deposits, Pump Date was weighted based on the expiration date and volume of each Deposit within the Donor Pool. Lactation Stage was computed based on Pump Date less Baby DOB and was reported in weeks postpartum. Milk type was assigned based on analysis of the dataset to determine when protein concentrations stabilized in the first 7 weeks postpartum. Based on this analysis, Transition Milk was defined as Lactation Stage less than or equal to four weeks and Mature Milk was defined as Lactation Stage between 4 and 52 weeks. A final category, Extended Milk, was classified as Donor Pools with a Lactation Stage greater than 52 weeks postpartum based on evidence that macronutrient composition changes in the second year postpartum [18]. Calorie content was calculated based on the USDA reference value for human milk using the following formula:

$$Calories\;(\frac{kcal}{dL})=Protein\;\left(\frac{g}{dL}\right)*4.4+Fat\;\left(\frac{g}{dL}\right)*8.79+Lactose\;\left(\frac{g}{dL}\right)*3.87$$

To analyze how fortification would affect the protein and calorie content of the Donor Pools, commercial fortifiers were identified and hypothetical post-fortification protein and energy content was calculated based on the manufacturer’s mixing instructions. Using the manufacturer’s mixing instructions and assuming milk composition of 1 g/dL protein, 7 g/dL lactose, 3.5 g/dL fat, and 19 kcal/ounce, Similac Human Milk Fortifier Concentrated Liquid (Abbott Laboratories, Chicago, IL) mixed to 24 kcal would increase protein by 1.0 g/dL and was considered a basic protein fortifier. Similac Human Milk Fortifier Hydrolyzed Protein Concentrated Liquid and Prolacta +6 (Prolacta Biosciences, City of Industries, CA) would increase protein by 1.5 g/dL and were considered intermediate protein fortifiers. Prolacta +10 (Prolacta Biosciences, City of Industries, CA) would increase protein by 2.5 g/dL and was considered a high protein fortifier [23,24]. Both Similac fortifiers would increase calories by 3.8 kcal/ounce and were considered a basic calorie fortifier. Prolacta +6 would increase calories by 6.8 kcal/ounce and was considered an intermediate calorie fortifier and Prolacta +10 was considered a high calorie fortifier, providing an extra 11.6 kcal/ounce. The macronutrient profile of each Donor Pool was used to compute feeding volumes necessary to achieve protein requirements of 3.5, 4.0, 4.5, and 5.0 g/kg/day using basic, intermediate, and high protein fortifiers. Similarly, feeding volumes necessary to achieve calorie targets of 110 and 135 kcal/kg/day were also computed. Feeding volumes were compared to two reference values: 200 ml/kg/day which is the upper range defined by ESPGHAN (10), and 160 ml/kg/day, which is a common goal volume used in United States NICUs [8,25–27]. Fortification and feeding volume calculations were performed independently by two researchers to ensure agreement. All identifying information was removed from the dataset prior to analysis.

Data were excluded for multiple reasons. For example, for some Donor Pools, the calculated Pump Date was earlier than the Infant DOB, likely due to data entry errors (n = 28; 1.5% of Donor Pools). For the purpose of maintaining conservative expiration dates, when milk is donated without a pump date on the label, MMBNT assigns the pump date as the infant DOB. For this reason, Donor Pools with a Pump Date equal to the Infant DOB were excluded from the analysis (n = 41; 2.2% of Donor Pools). Donor Pools from unique donors that had identical macronutrient values is suggestive of final pooling values versus individual donor pool values and were therefor excluded (n = 170; 9.0% of Donor Pools). To avoid masking effects associated with Stage of Lactation, Donor Pools that contained multiple Deposits for which the difference between earliest and latest Pump Dates was greater than 7 days were also excluded from the analysis (n = 537; 28.3% of Donor Pools).

### Statistical analysis

Statistical analysis was conducted in Minitab (Minitab Inc., State College, PA, USA), Microsoft Excel (Microsoft Corp., Redmond, WA, USA) and SAS Enterprise Edition 9.4 (SAS Corporation, Cary, NC, USA). The full dataset was used to describe prevalence of Donor Pools by Baby Term (Full, Preterm, Deceased) and by Lactation Stage (Transition, Mature, Extended), with differences in distribution evaluated using a Fisher’s Exact test. Descriptive statistics were computed on a subset of the data containing a single Donor Pool per donor in order not to violate statistical rules of independence between observations in a dataset. For donors with multiple Donor Pools, the first donation chronologically was used. Within this data subset, differences in Lactation Stage were evaluated using one-way ANOVA analysis with a Tukey test for multiple comparisons, and theoretical effects of fortification were explored. To explore subject versus time effects, a subset of the data containing donors with multiple Donor Pools was used. Coefficients of determination (R^2^) were calculated to determine the impact of Lactation Stage (a time-effect) and Donor ID (a subject-effect). The full dataset, which reflects the actual population of Donor Pools available for creating pooled donor human milk, was used to simulate random pooling of 1, 2, 3, 4, and 5 donors per pool. Briefly, the simulation involved confirming independence between deposit volume, protein, and fat and using historical data; generating values for these characteristics of each deposit independently using the empirical distributions; combining deposits into random pools using a variety of numbers of donors per pool; and calculating volume-weighted averages. Two-thousand random pools were generated for each number of donors in a pool (1 through 5). Simulated results were compared to historical data from MMBNT of pools created between November 2015 and July 2016 to target 19+ kcal/ounce using macronutrient profiles from individual Donor Pools. This research received an exempt status from the University of Pittsburgh Institutional Review Board (protocol #PR017070192).

## Results

The dataset included a total of 443 individual donors and 1,119 Donor Pools. Twenty-eight percent of the Donor Pools contained more than one Deposit. Eighty-five percent of the donors (375/443) and 84.4% (944/1,119) of the Donor Pools were from donors who had given birth at term. Donors of preterm infants represented 13.1% of donors (58/443) and 13.8% (154/1,119) of Donor Pools while bereaved donors of deceased infants represented 2.3% of donors (10/443) and 1.9% (21/1,119) of Donor Pools. There was a significant difference (P < 0.001) in the distribution of Donor Pools by Donor Term and Lactation Stage, with Term and Preterm donors providing mostly Mature Milk (75.9% and 64.3% of Donor Pools, respectively), while bereaved donors provided the highest percentage of Transition Milk (47.6% of Donor Pools). Forty-eight percent of the donors (214/443) had multiple Donor Pools in the dataset. The mean and median number of Donor Pools per donor were 2.5 and 2, respectively, while the maximum number of Donor Pools in the dataset from a single donor was 29.

### Macronutrient content by lactation stage

In a cross-sectional analysis of the initial Donor Pool from each unique donor (N = 443), 33.4% (148/443) were Transition Milk, 61.6% (273/443) were Mature Milk, and 5.0% (22/443) were Extended Milk. Protein concentration was different by Lactation Stage (P < 0.001), with Transition Milk having the highest protein (1.3 ± 0.2 g/dL) followed by Extended Milk (1.2 ± 0.2 g/dL) and Mature Milk (1.0 ± 0.2 g/dL). Fat and calories were significantly higher (P < 0.05) in Extended Milk (4.0 ± 1.0 g/dL and 20.0 ± 2.7 kcal/ounce) compared to Mature Milk (3.5 ± 0.9 g/dL and 18.7 ± 2.4 kcal/ounce). A summary of macronutrient composition by Lactation Stage is presented in Table 1.

**Table 1:** Descriptive statistics of macronutrients in human milk based on lactation stage from a cross-sectional analysis of 443 unique donors.

Lactation Stage:	Transition (n = 148)	Mature (n = 273)	Extended (n = 22)	P-value
Fat (g/dL)	3.5^ab^ (0.7)	3.5^a^ (0.9)	4.0^b^ (1.0)	0.061
Protein (g/dL)	1.3^a^ (0.2)	1.0^b^ (0.2)	1.2^c^ (0.2)	< 0.001
Lactose (g/dL)	7.2 (0.2)	7.2 (0.2)	7.2 (0.3)	0.432
Energy (kcal/oz)	19.2^ab^ (1.9)	18.7^a^ (2.4)	20.0^b^ (2.7)	0.013

Notes: Nutrient data represent mean and standard deviations. Differences by Lactation Stage were evaluated using a one-way ANOVA and Tukey test for multiple comparisons. Values with the same letter in the superscript are not statistically different (P > 0.05).

**Fig 1:**
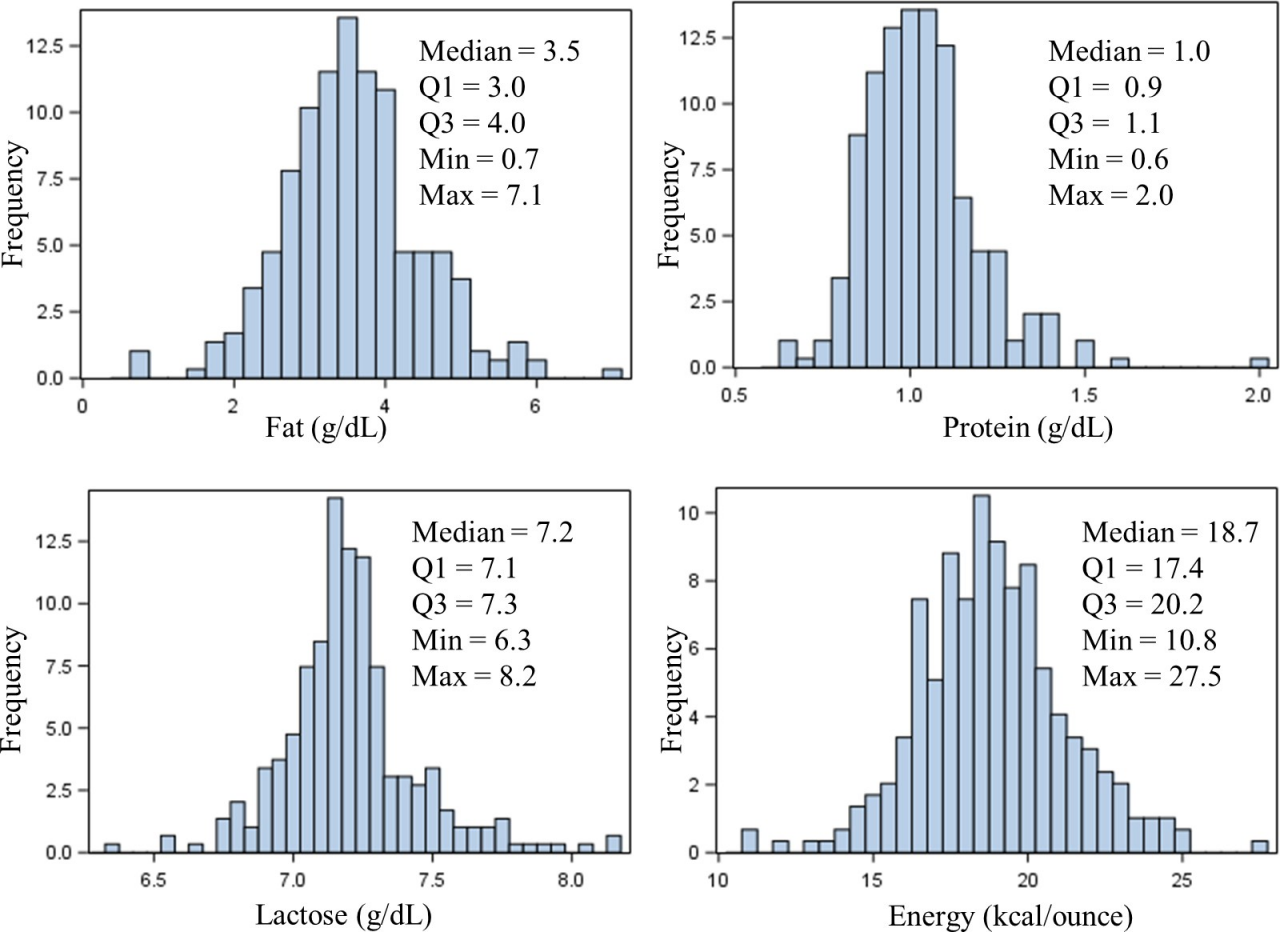
Histograms and descriptive statistics for donor pools. Represents Donor Pools > 4 weeks postpartum from 295 unique donors.

The distribution of macronutrients in the initial Donor Pools with a Lactation Stage greater than 4 weeks from 295 unique donors is presented in histogram format (Fig 1), along with median and quartile values. There was a 33% difference in fat content between quartile 1 (3.0 g/dL) and quartile 3 (4.0 g/dL), which represents a calorie difference of 2.7 kcal/ounce; a 22% difference (0.2 kcal/ounce) in the protein content between quartile 1 (0.9 g/dL) and quartile 3 (1.1 g/dL); a 3% difference (0.2 kcal/ounce) in the lactose content between quartile 1 (7.1 g/dL) and quartile 3 (7.3 g/dL); and a 16% difference in the energy content between quartile 1 (17.4 kcal/ounce) and quartile 3 (20.2 kcal/ounce).

### Meeting protein and calorie requirements

**Fig 2:**
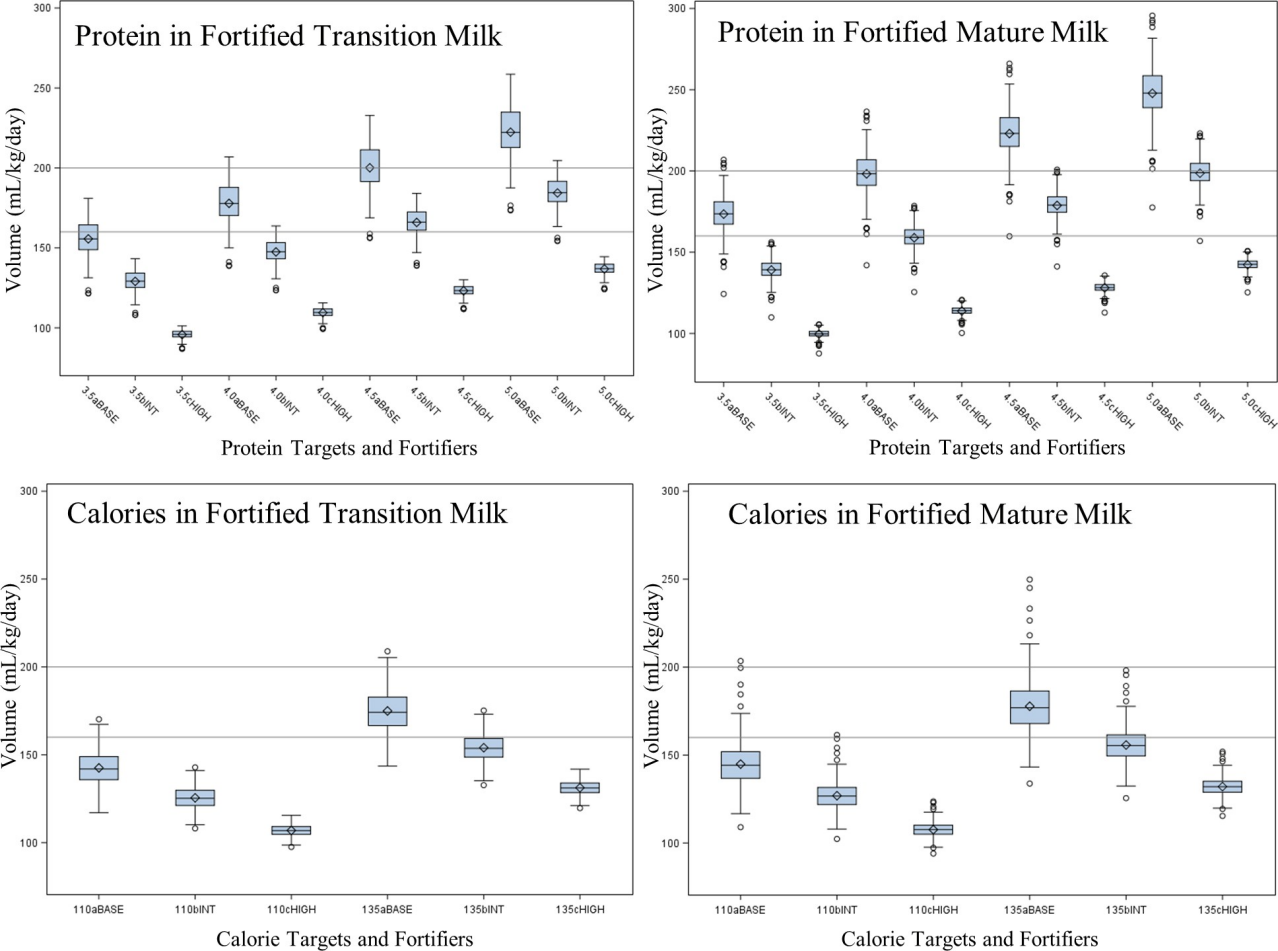
Box and whisker plots of feeding volumes to achieve various protein and calorie targets using commercial fortifiers. Base (BASE), intermediate (INT), and high (HIGH) protein and calorie fortifiers in 443 Donor Pools from 443 unique donors providing either milk < = 4 weeks postpartum (N = 148) or > 4 weeks postpartum (N = 295). Grey rectangles represent quartile 1 to quartile 3 values. Within rectangles, the median is represented by–and the mean is represented by ◊. Reference lines: 200 ml/kg/day represents ESPGHAN maximum feeding volume; 160 ml/kg/day represents common NICU target volume.

In unfortified milk, the median feeding volumes to reach protein targets of 3.5 to 5.0 g/kg ranged from 269 to 490 ml/kg of body weight, with lower volumes required for Transition Milk compared to Mature Milk due to higher protein content. Median daily feeding requirements ranged from 156 to 248 ml/kg with basic protein fortification, 129 to 199 ml/kg with intermediate protein fortification, and 96 to 143 ml/kg with high protein fortification. Box and whisker plots illustrate the distribution of feeding volumes required to reach various protein and calorie targets using fortified Transition and Mature milk (Fig 2).

### Subject versus time effect

Only donors with multiple Donor Pools were included in a bivariate analysis to explore the relationship between macronutrients and Lactation Stage (a time-effect) versus Donor ID (a subject-effect). When including all Lactation Stages, the subject-effect explained more of the macronutrients (R^2^ values ranging from 0.511 to 0.828) than the time-effect (R^2^ values ranging from 0.105 to 0.492). When excluding Transition Milk where significant temporal changes are expected, the difference between the subject-effect (R^2^ values ranging from 0.507 to 0.848) and the time effect (R^2^ values ranging from 0.100 to 0.278) were even more pronounced. Results are summarized in Table 2.

**Table 2:** Impact of subject-effect versus time-effect on predicting macronutrients in donors with multiple donor pools.

	Coefficients of Determination (R^2^) for Donors with Multiple Donor Pools			
	All Donor Pools		Donor Pools Excluding Transition Milk	
	Subject Effect	Time Effect	Subject Effect	Time Effect
# Donors	232	232	214	214
# Deposits	890	890	706	706
Protein	0.699	0.492	0.770	0.278
Fat	0.828	0.161	0.848	0.182
Lactose	0.511	0.105	0.507	0.100

Note: Data represent Coefficients of Variation (R^2^) from a bivariate analysis of subject and time effect on macronutrient composition.

### Impact of nutrient variability on pooled donor milk

**Fig 3:**
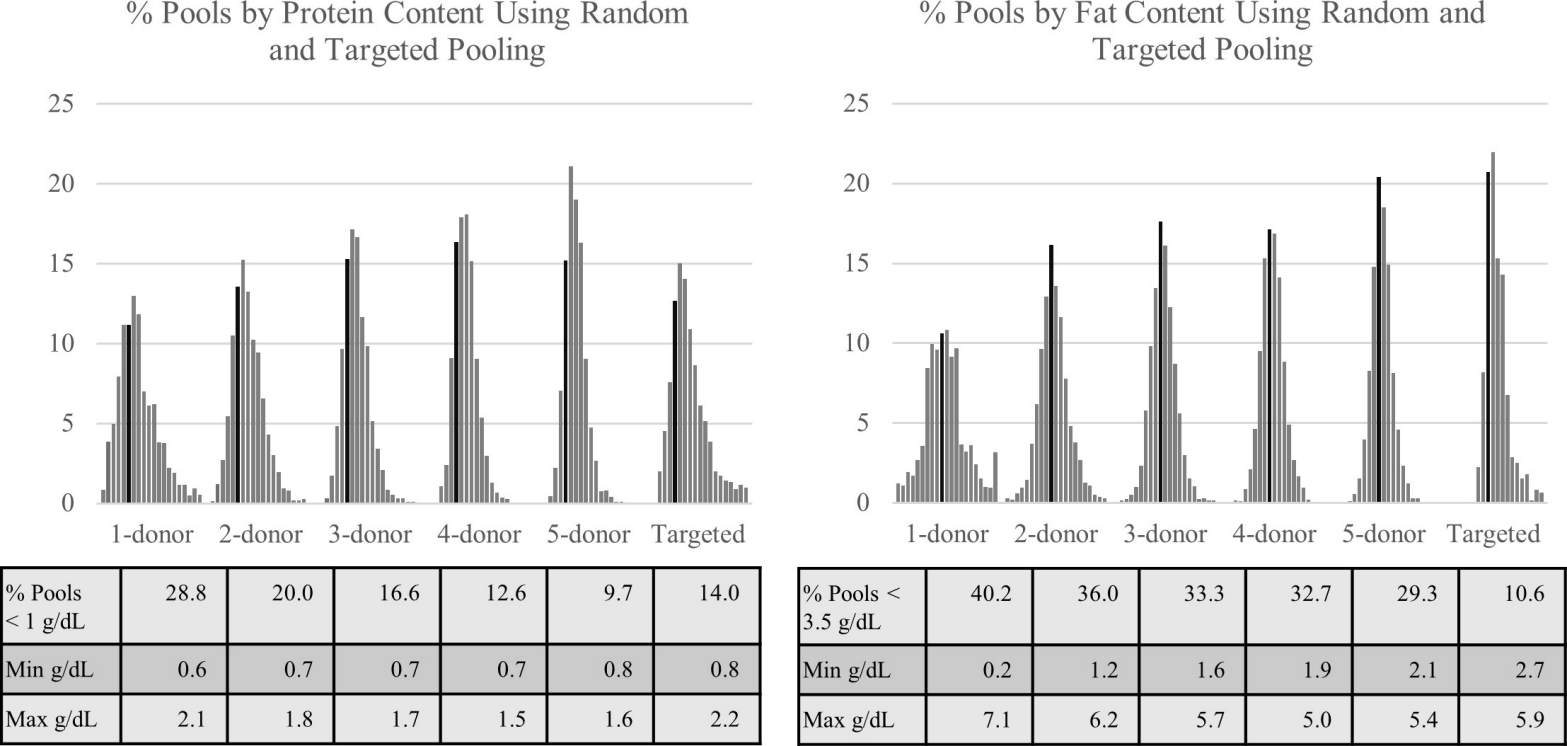
Percent of pools by protein and fat content based on random pooling of 1 to 5 donors, or targeted pooling. Dark bar represents percent of pools containing 1.0 g/dL of protein and 3.5 g/dL of fat.

Milk banks in North America typically pool the milk from 2–5 individual donors in order to reduce the nutrient variability in pooled donor milk [20]. We simulated random pooling of 1, 2, 3, 4, and 5 donors to assess the impact of pooling practice. Results of 2,000 randomly simulated pools at 5 different donor levels per pool (1 to 5 donors) are presented in Fig 3. As expected, as the number of donors in a random pool increased, the range of the nutrients in the simulated pools decreased. Using 1 g/dL protein and 3.5 g/dL fat as target threshold based on commonly reported averages for human milk, 28.8% of 1-donor pools fell below 1 g/dL protein, compared to 9.7% of randomly generated 5-donor pools. Similarly, 40.2% of 1-donor pools fell below 3.5 g/dL of fat, compared to 29.3% of randomly generated 5-donor pools.

Some milk banks, including MMBNT, create targeted pools of donor milk based on macronutrient analysis of individual Donor Pools. The historical targeted pooling data from MMBNT represented 1,686 batches of donor milk produced between November 2015 and July 2016. Milk that was target pooled to 19–20 kcal/ounce represented 55.7% (939/1686) of the production; high calorie milk (> 22 kcal/ounce) represented 10.2% (172/1686) of the production; and outpatient/skim/dairy-free milk represented 34.1% (575/1686) of the production. A distribution of the fat and protein content of MMBNT’s 1,111 pools intended for the NICU (19+ kcal) is presented in Fig 3. The percentage of targeted pools below 1 g/dL of protein and 3.5 g/dL of fat were 14.0% and 10.6%, respectively.

## Discussion

Temporal changes in human milk composition are well-defined in the literature, with rapid changes observed in the first few weeks postpartum, as milk moves from colostrum to mature milk [12,13,16,28,29]. During this transition to mature milk, protein declines, while fat and lactose concentrations increase. These trends have been observed in women delivering at term, and women delivering prematurely, though preterm milk has been to shown to have higher initial protein composition than term milk, with the largest differences observed in the first few weeks postpartum [14,15,28,29]. The dataset in this study captured the significant decline in protein composition in the first 4 weeks postpartum, but did not capture increases in fat and lactose. The magnitude of protein changes in the early postpartum period are large compared to the magnitude of changes in lactose and fat [16], and smaller changes may have been masked by the cross-sectional nature of this study as well as the range of pumping dates included in each Donor Pool. A higher protein concentration associated with gradual weaning has been described by others in the second year postpartum [18,30,31], and are supported by our analysis.

The ability of Stage of Lactation (a time-effect) to predict the macronutrient composition of milk was small compared to the usefulness of Donor ID (a subject-effect) in predicting nutrient composition for all macronutrients in Transition and Mature Milk. This suggests that average values often assumed for human milk composition may not be representative of an individual’s milk composition. Others have reported a high inter-individual variability in the macronutrient composition in human milk [12,18,32–35]. Michaelson et al. analyzed 2554 milk samples from 224 mothers and found a 48% difference in fat, a 27% difference in protein, and a 5% difference in carbohydrates between quartile 1 and quartile 3 [12]. In our study there was a 33% difference in the fat content between the quartile 1 mark (3.0 g/dL) and the quartile 3 mark (4.0 g/dL) which translates into a difference of 2.7 kcal/ounce attributed to fat. A quarter of the Donor Pools contained 3.0 g/dL or less of fat. Evidence suggests that multiple external factors impact the fat composition of human milk, including maternal diet, maternal parity, and when the milk was pumped, which likely contributes to the high variability observed in fat composition [16,17,36–39].

Understanding how the natural variability in human milk composition contributes to the ability to meet nutrient needs in the NICU is an important area of research. Koo et al. examined the impact of multiple commercial fortifiers in meeting preterm infant requirements and reported that macronutrient recommendations were typically met; however, they used “average” human milk values cited by the manufacture and did not consider the impact of individual variation [40]. In our dataset of human milk from over 400 individuals, basic protein fortification could not achieve protein targets of 3.5 g/kg in more than 25% of transition milk samples, and over 75% of mature milk samples while staying below feeding volumes of 160 ml/kg. Intermediate protein fortification was able to meet protein targets of 4.0 g/kg in over 75% of transition milk samples and approximately 50% of mature milk samples at daily feeding volumes below 160 ml/kg. When protein targets increase to 4.5 g/kg, feeding volumes must exceed 160 ml/kg in the majority of samples using intermediate protein fortifiers. High protein fortification could meet all protein targets in all samples at feeding volumes below 160 ml/kg. Higher calorie targets (135 kcal/kg) were not met in over 75% of samples with basic calorie fortification, and approximately 25% of samples with intermediate calorie fortification, while staying within daily feeding volumes of 160 ml/kg.

The impact of human milk macronutrient variability on pooled donor milk is not well understood. Smith et al. reported creamatocrit values ranged from 1.7 to 7.3% in 22 samples of pooled donor milk from 3 to 4 donors [41]. A study of 33 pooled donor milk samples from 51 donors reported that fat was the most variable nutrient, with a coefficient of variation of 49%, though number of donors per pool was not reported [18]. In our simulation of randomly pooled donors, the number of donors included in each pool increased the percentage of pools reaching target fat and protein values. Targeted pooling based on macronutrient analysis of Donor Pools was especially effective at achieving a minimum level of fat in pooled donor milk. Each 1 gram/dL decrease in the fat content of human milk translates into a reduction of 14.4 kcal/kg/day at feeding volumes of 160 ml/kg/day. Energy intake in the preterm population is significantly associated with weight, length, and head growth [42]. Given that fat is a major contributor to the energy content in donor milk, reducing this variability is warranted when feeding nutritionally at-risk populations.

### Limitations

The dataset represents primarily milk from women who delivered at term. In the NICU environment when feeding infants with mother’s own milk, we would expect higher protein content in the milk of mothers delivering preterm. This would translate into lower feeding volumes to reach protein targets. A systematic review of preterm and term milk composition found that the average protein difference was only 0.2 g/dL by the second week postpartum [14], which would translate into volume reductions of 17 to 23 ml/kg/day over those reported in this study. This suggests that many samples, even when adjusted for higher protein levels expected in preterm milk, would require more than 150 to 160 ml/kg/day of feeding volumes in order to meet high protein targets. Donor Pools represented milk from a range of donors at each lactation stages; therefore, some temporal changes that occur in the early postpartum period, including an increase in fat and lactose, were not observed in this dataset. Historical results from targeted pooling are based on MMBNT production dates, while the Donor Pools for the simulation are based on receipt data; therefore, not all Donor Pools from the simulation may be represented in the historical targeted pooling results. However, the large dataset sizes and extensive date ranges contribute to a representative sample.

## Conclusions

The macronutrient profile of human milk varies significantly by subject, with differences in fat composition explaining up to 80% of the difference in calories. Individual variability can impact nutrients in pooled donor milk. This variability can be reduced by increasing the number of donors in a pool or by target pooling. While protein is frequently considered a rate limiting nutrient for preterm infant growth, providing adequate total calories is also an important consideration in the NICU. Basic protein fortification of human milk typically requires feeding volumes above 160 ml/kg to reach protein targets of 3.5 to 4.0 g/kg. There is an urgent need for research into how the nutrient variability in human milk impacts preterm infant growth, body composition, and short-term and long-term health outcomes.

## Supporting information

S1 File Macronutrient Data for Donor Pools and Historical Target Pools. (XLSX)

## Abbreviations

DOB: date of birth
HMBANA: Human Milk Banking Association of North America
MMBNT: Mothers’ Milk Bank North Texas
NEC: necrotizing enterocolitis
NICU: neonatal intensive care unit
VLBW: very low birth weight

## References

[1] Ong KK, Kennedy K, Castañeda-Gutiérrez E, Forsyth S, Godfrey KM, Koletzko B, et al Postnatal growth in preterm infants and later health outcomes: a systematic review. Acta Paediatr. 2015;104(10):974–86. 10.1111/apa.13128 26179961 PMC5054880

[2] Stephens BE, Walden RV, Gargus RA, Tucker R, McKinley L, Mance M, et al First-week protein and energy intakes are associated with 18-month developmental outcomes in extremely low birth weight infants. Pediatrics. 2009;123(5):1337–43. 10.1542/peds.2008-0211 19403500

[3] Christmann V, Roeleveld N, Visser R, Janssen AJW., Reuser JJC., Goudoever JB, et al The early postnatal nutritional intake of preterm infants affected neurodevelopmental outcomes differently in boys and girls at 24 months. Acta Paediatr. 2017;106(2):242–9. 10.1111/apa.13669 27862266 PMC5248638

[4] Ehrenkranz RA, Das A, Wrage LA, Poindexter BB, Higgins RD, Stoll BJ, et al Early nutrition mediates the influence of severity of illness on extremely LBW infants. Pediatr Res. 2011;69(6):522–9. 10.1203/PDR.0b013e318217f4f1 21378596 PMC3090495

[5] American Academy of Pediatrics, Section on Breastfeeding. Breastfeeding and the use of human milk. Pediatrics. 2012;129(3):827–41.

[6] Ziegler EE. Meeting the Nutritional Needs of the Low-Birth-Weight Infant. Ann Nutr Metab. 2011;58(Supplement):8–18. 10.1159/000323381 21701163

[7] Quigley M, McGuire W. Formula versus donor breast milk for feeding preterm or low birth weight infants. Cochrane Database Syst Rev. 2014 4 22;(4):CD002971 10.1002/14651858.CD002971.pub3 24752468

[8] Sisk PM, Lambeth TM, Rojas MA, Lightbourne T, Barahona M, Anthony E, et al Necrotizing Enterocolitis and Growth in Preterm Infants Fed Predominantly Maternal Milk, Pasteurized Donor Milk, or Preterm Formula: A Retrospective Study. Am J Perinatol. 2016 12 9; 10.1055/s-0036-1597326 27936476

[9] Perrin MT. Donor Human Milk and Fortifier use in United States level 2, 3, and 4 Neonatal Care Hospitals. J Pediatr Gastroenterol Nutr. 2017; 10.1097/MPG.0000000000001790 29045350

[10] Agostoni C, Buonocore G, Carnielli VP, De Curtis M, Darmaun D, Decsi T, et al Enteral nutrient supply for preterm infants: commentary from the European Society of Paediatric Gastroenterology, Hepatology and Nutrition Committee on Nutrition. J Pediatr Gastroenterol Nutr. 2010;50(1):85–91. 10.1097/MPG.0b013e3181adaee0 19881390

[11] Koletzko B, Poindexter B, Uauy R. Nutritional care of preterm infants: scientific basis and practical guidelines [Internet]. 2014. Available from: http://public.eblib.com/choice/publicfullrecord.aspx?p=3016615

[12] Michaelsen KF, Skafte L, Badsberg JH, Jørgensen M. Variation in macronutrients in human bank milk: influencing factors and implications for human milk banking. J Pediatr Gastroenterol Nutr. 1990;11(2):229–39. 2395063 10.1097/00005176-199008000-00013

[13] Allen JC, Keller RP, Archer P, Neville MC. Studies in human lactation: milk composition and daily secretion rates of macronutrients in the first year of lactation. Am J Clin Nutr. 1991;54(1):69–80. 10.1093/ajcn/54.1.69 2058590

[14] Gidrewicz DA, Fenton TR. A systematic review and meta-analysis of the nutrient content of preterm and term breast milk. BMC Pediatr. 2014;14. 10.1186/1471-2431-14-216 PMC4236651 25174435

[15] Boyce C, Watson M, Lazidis G, Reeve S, Dods K, Simmer K, et al Preterm human milk composition: a systematic literature review. Br J Nutr. 2016;116(6):1033–45. 10.1017/S0007114516003007 27522863

[16] Saarela T, Kokkonen J, Koivisto M. Macronutrient and energy contents of human milk fractions during the first six months of lactation. Acta Paediatr. 2005;94(9):1176–81. 10.1080/08035250510036499 16203669

[17] Khan S, Hepworth AR, Prime DK, Lai CT, Trengove NJ, Hartmann PE. Variation in fat, lactose, and protein composition in breast milk over 24 hours: associations with infant feeding patterns. J Hum Lact. 2013;29(1):81–9. 10.1177/0890334412448841 22797414

[18] Perrin MT, Fogleman AD, Newburg DS, Allen JC. A longitudinal study of human milk composition in the second year postpartum: implications for human milk banking. Matern Child Nutr. 2017;13(1). 10.1111/mcn.12239 PMC6866067 26776058

[19] Horbar JD, Ehrenkranz RA, Badger GJ, Edwards EM, Morrow KA, Soll RF, et al Weight Growth Velocity and Postnatal Growth Failure in Infants 501 to 1500 Grams: 2000–2013. Pediatr. 2015;136(1):e84–92. 10.1542/peds.2015-0129 26101360

[20] Human Milk Banking Association of North America. Guidelines for the estalishment and operations of a donor human milk bank. 2018.

[21] Michaelsen KF, Pedersen SB, Skafte L, Jaeger P, Peitersen B. Infrared analysis for determining macronutrients in human milk. J Pediatr Gastroenterol Nutr. 1988;7(2). 10.1097/00005176-198803000-00013 3351708

[22] Fusch G, Kwan C, Kotrri G, Fusch C. “Bed Side” Human Milk Analysis in the Neonatal Intensive Care Unit: A Systematic Review. Clin Perinatol. 2017;44(1):209–67. 10.1016/j.clp.2016.11.001 28159207

[23] Similac Human Milk Fortifier Concentrated Liquid [Internet]. Available from: https://abbottnutrition.com/similac-human-milk-fortifier-concentrated-liquid

[24] Prolacta Bioscience [Internet]. [cited 2017 Jan 13]. Available from: http://www.prolacta.com/home

[25] Hair AB, Peluso AM, Hawthorne KM, Perez J, Smith DP, Khan JY, et al Beyond Necrotizing Enterocolitis Prevention: Improving Outcomes with an Exclusive Human Milk-Based Diet. Breastfeed Med. 2016;11(2):70–4. 10.1089/bfm.2015.0134 26789484 PMC4782036

[26] Assad M, Elliott MJ, Abraham JH. Decreased cost and improved feeding tolerance in VLBW infants fed an exclusive human milk diet. J Perinatol. 2016;36(3):216–20. 10.1038/jp.2015.168 26562370

[27] Baylor College of Medicine. Guidelines for Acute Care of the Neonate, Edition 25 2017–2018 [Internet]. Section of Neonatology, Department of Pediatrics; 2017 [cited 2018 Jan 31]. Available from: https://www.bcm.edu/departments/pediatrics/sections-divisions-centers/neonatology/publications/physician

[28] Narang APS, Bains HS, Kansal S, Singh D. Serial composition of human milk in preterm and term mothers. Indian J Clin Biochem. 2006;21(1):89–94. 10.1007/BF02913072 23105575 PMC3453757

[29] Bauer J, Gerss J. Longitudinal analysis of macronutrients and minerals in human milk produced by mothers of preterm infants. Clin Nutr Edinb Scotl. 2011;30(2):215–20. 10.1016/j.clnu.2010.08.003 20801561

[30] Garza C, Johnson CA, Smith EO, Nichols BL. Changes in the nutrient composition of human milk during gradual weaning. Am J Clin Nutr. 1983;37(1):61–5. 10.1093/ajcn/37.1.61 6849283

[31] Neville MC, Allen JC, Archer PC, Casey CE, Seacat J, Keller RP, et al Studies in human lactation: milk volume and nutrient composition during weaning and lactogenesis. Am J Clin Nutr. 1991;54(1):81–92. 10.1093/ajcn/54.1.81 2058592

[32] Góes HC., Torres AG, Donangelo CM, Trugo NM. Nutrient composition of banked human milk in brazil and influence of processing on zinc distribution in milk fractions. Nutr. 2002;18(7):590–4. 10.1016/s0899-9007(02)00813-4 12093436

[33] Wojcik KY, Rechtman DJ, Lee ML, Montoya A, Medo ET. Macronutrient Analysis of a Nationwide Sample of Donor Breast Milk. J Am Diet Assoc. 2009 1;109(1):137–40. 10.1016/j.jada.2008.10.008 19103335

[34] Cooper AR, Barnett D, Gentles E, Cairns L, Simpson JH. Macronutrient content of donor human breast milk. Arch Dis Child Fetal Neonatal Ed. 2013;98(6):539–41. 10.1136/archdischild-2013-304422 23867707

[35] de Halleux V, Rigo J. Variability in human milk composition: benefit of individualized fortification in very-low-birth-weight infants. Am J Clin Nutr. 2013;98(2). 10.3945/ajcn.112.042689 23824725

[36] Yahvah KM, Brooker SL, Williams JE, Settles M, McGuire MA, McGuire MK. Elevated dairy fat intake in lactating women alters milk lipid and fatty acids without detectible changes in expression of genes related to lipid uptake or synthesis. Nutr Res. 2015;35(3):221–8. 10.1016/j.nutres.2015.01.004 25661476

[37] Mohammad MA, Sunehag AL, Haymond MW. Effect of dietary macronutrient composition under moderate hypocaloric intake on maternal adaptation during lactation. Am J Clin Nutr. 2009;89(6):1821–7. 10.3945/ajcn.2008.26877 19386740 PMC2682997

[38] Bachour P, Yafawi R, Jaber F, Choueiri E, Abdel-Razzak Z. Effects of smoking, mother’s age, body mass index, and parity number on lipid, protein, and secretory immunoglobulin A concentrations of human milk. Breastfeed Med. 2012;7(3):179–88. 10.1089/bfm.2011.0038 22166069

[39] Bravi F, Wiens F, Decarli A, Pont AD, Agostoni C, Ferraroni M. Impact of maternal nutrition on breast-milk composition: a systematic review. Am J Clin Nutr. 2016;104(3):646 10.3945/ajcn.115.120881 27534637

[40] Koo W, Tice H. Human Milk Fortifiers Do Not Meet the Current Recommendation for Nutrients in Very Low Birth Weight Infants. J Parenter Enteral Nutr. 2017;2017:148607117713202. 10.1177/0148607117713202 28622483

[41] Smith L, Harkes A, D’Souza SW. Fat content and fatty acid composition of pooled banked milk. Br Med J Clin Res Ed. 1984 1 28;288(6413):283. 10.1136/bmj.288.6413.283 PMC1444034 6419895

[42] Stoltz Sjöström E, Ohlund I, Ahlsson F, Engstrom E, Fellman V, Hellstrom A, Kallen K, Norman M, Olhager E, Serenius F, Dommelof M. Nutrient intakes independently affect growth in extremely preterm infants: results from a population-based study. *Acta Pædiatrica* 102, 1067–1074 (2013) 10.1111/apa.12359 23855971

